# Dopant-Engineered Downshifting Nanoparticles with Dual NIR-II Fluorescence and Magnetic Resonance Imaging for Diagnosis and Image-Guided Surgery of Breast Cancer

**DOI:** 10.3390/bios16030180

**Published:** 2026-03-23

**Authors:** Zia Ullah, Mu Du, Lihong Jiang, Yibin Yan, Yuqian Yan, Jingsi Gu, Jing Cheng, Bing Guo, Zun Wang

**Affiliations:** 1 School of Science, Harbin Institute of Technology, Shenzhen 518055, China; 2 Department of Radiology, Southern University of Science and Technology Hospital, Shenzhen 518055, China; 3 Education Center of Experiments and Innovations, Harbin Institute of Technology, Shenzhen 518055, China; 4 Department of Breast and Thyroid Surgery, Shenzhen Baoan Women’s and Children’s Hospital, Jinan University, Shenzhen 518000, China

**Keywords:** downshifting nanoparticles, NIR-II fluorescence imaging, magnetic resonance imaging, breast cancer, imaging-guided surgery

## Abstract

As surgery is the first-line paradigm for many solid tumors, precision in preoperative diagnosis and intraoperative imaging is of significant importance. Dual MRI and NIR-II fluorescence imaging could fulfill precision imaging requirements in treating cancers, because of its deep penetration and real-time high spatiotemporal resolution. Thus, the design of dual MRI/NIR-II fluorescence contrast agents is crucial for the diagnosis and surgery of cancers. Herein, we developed optically transparent NaGdF4 matrix-based downshifting nanoparticles (DSNPs) co-doped with Nd^3+^, Yb^3+^, and Er^3+^ as a single nanoplatform for dual NIR-II fluorescence and T1-weighted MRI. Systematic dopant engineering reveals that optimal Nd^3+^ loading enhances cascade Nd → Yb → Er energy transfer and yields intense NIR-II emission at 1334 and 1521 nm upon 808 nm excitation with a relative quantum yield of 1.55, while the presence of Gd^3+^ in the optically transparent matrix imparts strong T1 contrast (4.98 s^−1 ^mM^−1^). The Pluronic F-127 surface coating confers colloidal stability and biocompatibility. In vitro assays confirm negligible cytotoxicity and efficient cellular uptake. In vivo studies in subcutaneous 4T1 tumor-bearing mice demonstrate robust accumulation, high tumor-to-background contrast in both MRI/NIR-II fluorescence and enable precise NIR-II fluorescence imaging-guided surgery with real-time margin visualization. Therefore, dopant-engineered DSNPs represent a promising dual-modal imaging agent for deep-tissue diagnostic and real-time surgical guidance in precision oncology.

## 1. Introduction

Cancer is one of the leading causes of mortality worldwide, and solid tumors account for the majority of cancer-caused deaths [1]. The improvement in patient prognosis heavily relies on precise imaging technologies that can enable accurate tumor localization, complete tumor resection, and effective postoperative monitoring [2]. Clinical imaging plays a central role in this process by providing anatomical, functional, and molecular information that guides therapeutic planning [3,4]. Magnetic resonance imaging (MRI) has emerged as one of the most important diagnostic tools due to its excellent soft-tissue contrast, deep-tissue penetration, and lack of ionizing radiation among the available clinical imaging modalities [5]. MRI is widely used for tumor detection, staging, and preoperative planning [6]. However, surgery remains the first-line treatment for most solid tumors because complete tumor resection with clear margins is directly associated with improved survival outcomes [7]. In this context, intraoperative fluorescence (FL) imaging-guided surgery has attracted significant attention as it enables real-time visualization of tumor boundaries and assists the surgeons in achieving maximal tumor removal while sparing healthy tissue [8,9]. Therefore, high precision in both preoperative diagnosis and intraoperative imaging guidance is crucial for improving surgical accuracy and overall treatment outcomes.

In clinical settings, different imaging agents are routinely used for preoperative MRI and intraoperative FL imaging-guided surgery [10]. Water-soluble Gd-based contrast agents like gadoterate meglumine and gadobutrol are commonly administered intravenously to enhance lesion visibility and tissue contrast for MRI examination [11]. On the other hand, small-molecule fluorescent dyes such as sodium fluorescein and indocyanine green (ICG) are frequently employed to assist tumor visualization during surgical procedures [12,13]. These agents are effective in their respective applications but exhibit different targeting behavior, pharmacokinetics, and pharmacodynamics [14]. That is why the tumor localization observed during preoperative MRI often does not fully correlate with the FL distribution during surgery and can potentially lead to discrepancies between diagnostic imaging and intraoperative guidance [15]. Moreover, the rapid renal clearance and short circulation times of small-molecule imaging agents limit their tumor retention and thus they require repeated administrations [16]. Therefore, an ideal imaging strategy would employ a single contrast agent that can provide both MRI and FL imaging with consistent tumor targeting, pharmacokinetics, and biodistribution. More importantly, the synergistic integration of MRI and second near-infrared (NIR-II) FL imaging holds particular promise for precision oncology [17]. MRI offers deep penetration and high-resolution anatomical mapping; on the other hand, NIR-II FL imaging enables real-time imaging with superior spatial and temporal resolution, reduced photon scattering, and minimal tissue autofluorescence [18]. Dual-modal MR and NIR-II FL imaging could fulfill the stringent requirements of precision cancer imaging by combining deep-tissue visualization with high-contrast and real-time intraoperative guidance [18].

Despite the conceptual appeal of dual-modal MR and NIR-II FL imaging, the development of practical dual-modal MRI contrast agents and NIR-II fluorescent probes remains challenging. Clinically used water-soluble imaging agents exhibit favorable renal clearance and short-term safety profiles [19,20]. However, their rapid diffusion and limited retention at tumor sites restrict their utility during prolonged surgical procedures [21]. Physical disruption of tumor tissue and the surrounding vasculature can destroy physiological and pathological barriers, allowing small-molecule dyes to diffuse uncontrollably into normal tissues and blood vessels during surgery [22]. This non-specific distribution generates background signals that interfere with tumor delineation and compromise surgical precision [23]. In contrast, nanoparticle-based imaging agents offer distinct advantages for integrated preoperative diagnosis and intraoperative guidance [24]. These nanoparticle-based imaging agents can exploit the enhanced permeability and retention (EPR) effect for preferential tumor accumulation and can be rationally engineered in terms of size, shape, and surface chemistry to modulate biodistribution, circulation time, and tumor retention [6,25]. To date, nanoparticle-based MRI contrast agents and NIR-II fluorescent probes have included inorganic nanomaterials, organic/inorganic hybrids, and polymer-based systems [8,26]. However, these platforms suffer from limited photostability, insufficient NIR-II emission efficiency, suboptimal MRI contrast, and long-term biosafety concerns. This highlights the need for more robust and clinically translatable solutions.

Rare-earth-doped downshifting nanoparticles (DSNPs) have emerged as an attractive class of nanomaterials for dual-modal MR and NIR-II FL imaging. DSNPs possess unique optical properties because of their ladder-like 4f electronic energy levels, enabling anti-Stokes emission, narrow and tunable emission bands, and exceptional photostability [27]. DSNPs are resistant to photobleaching and chemical degradation as compared to organic fluorophores and conjugated polymers, which makes them suitable for prolonged imaging applications. DSNPs also avoid heavy metal toxicity concerns and exhibit well-defined emission characteristics, unlike quantum dots and carbon-based nanomaterials [28]. Moreover, DSNPs can be intrinsically engineered to serve as MRI contrast agents by incorporating paramagnetic ions such as Fe^3+^, Mn^2+^, and Gd^3+^ into the host lattice, thereby enabling simultaneous optical and magnetic imaging within a single nanoplatform [29]. Nevertheless, the optimization of DSNPs for dual-modal imaging is nontrivial because the interionic energy transfer processes and concentration-dependent quenching effects must be carefully balanced [11]. In particular, the interactions between sensitizer ions and activator ions play a decisive role in determining NIR-II emission efficiency. Excessive dopant concentrations can degrade both optical and magnetic performance [30]. Moreover, systematic studies linking dopant engineering, multimodal imaging performance, and in vivo surgical applicability remain limited. The incorporation of paramagnetic ions like Fe^3+^, Mn^2+^, and Gd^3+^ into the host lattice can hamper the NIR-II emission efficiency by decreasing the optical transparency of the matrix [19]. Therefore, the incorporation of magnetic properties and FL properties in the optically transparent matrix could be an ideal strategy for designing dual-modal MRI contrast agents and NIR-II fluorescent probes.

**Scheme 1:**
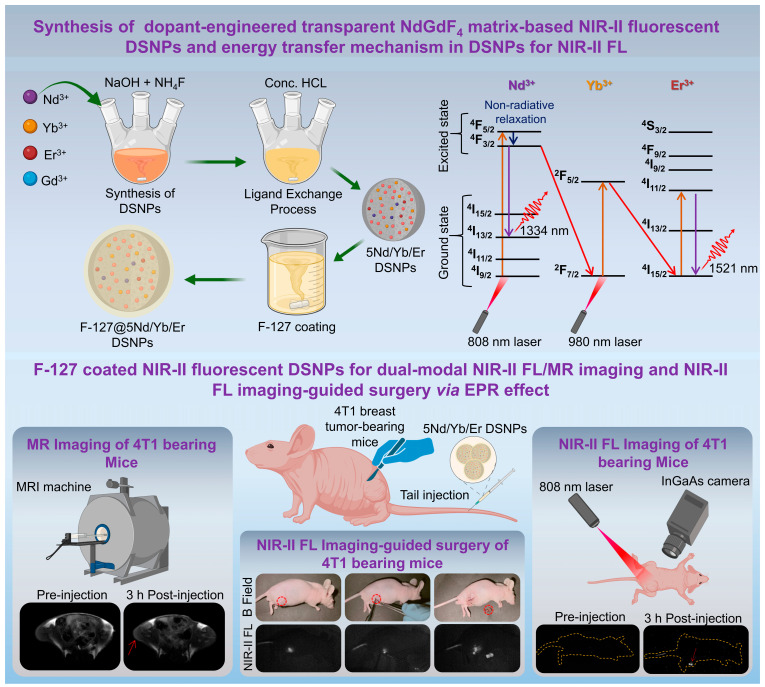
Schematic representation of the synthesis, cascade energy transfer mechanism, and in vivo dual-modal MR and NIR-II FL imaging application of dopant-engineered 5Nd/Yb/Er DSNPs. The optically transparent NaGdF_4_ matrix-based DSNPs co-doped with (2.5–7.5%) Nd^3+^, (25%) Yb^3+^, and (5%) Er^3+^ were synthesized and followed by ligand exchange and surface functionalization with biocompatible Pluronic F-127 to improve aqueous dispersibility and biological stability. The energy transfer mechanism under 808 nm excitation shows that Nd^3+^ acts as the primary sensitizer, transferring energy to Yb^3+^ and subsequently to Er^3+^, resulting in characteristic NIR-II FL emissions at 1334 nm and 1521 nm. The presence of Gd^3+^ in the optically transparent NaGdF_4_ host lattice provides T1-weighted MRI contrast. The in vivo administration of the dopant-engineered 5Nd/Yb/Er DSNPs in subcutaneous 4T1 breast tumor-bearing mice for dual-modal MR and NIR-II FL imaging and real-time NIR-II FL imaging-guided surgical resection demonstrates their application in precise tumor delineation and imaging-guided surgical intervention. Red circles and arrows indicate the tumor site.

Building on this, we designed optically transparent NdGdF_4_ matrix-based DSNPs co-doped with Nd^3+^ (2.5–7.5%), Yb^3+^ (25%), and Er^3+^ (5%) as a dual-modal imaging nanoplatform for NIR-II FL and MR imaging and demonstrated their efficacy in NIR-II FL imaging-guided surgery of subcutaneous 4T1 breast tumor-bearing mice (Scheme 1). For optimizing the imaging performance, we systematically engineered the Nd^3+^ dopant concentration while keeping the Yb^3+^ and Er^3+^ loading fixed. This enabled a detailed elucidation of the dopant engineering on energy transfer pathways and multimodal imaging characteristics. Comprehensive optical studies revealed that optimized Nd^3+^ doping (5%) markedly enhanced absorption in the NIR region, facilitated efficient cascade Nd → Yb → Er energy transfer, and yielded intense NIR-II emissions at 1334 nm and 1521 nm upon 808 nm excitation with a relative quantum yield (Φ) of 1.55. In parallel, the presence of Gd^3+^ in the optically transparent NaGdF_4_ host lattice endowed the DSNPs with significant T1 relaxivity of 4.98 s^−1^ mM^−1^ and affirmed their capability as efficient MR contrast agents. To improve physiological stability and biocompatibility, the optimized DSNPs were coated with a biocompatible Pluronic F-127 polymer that confers excellent colloidal stability in aqueous media and controls non-specific interactions [8]. In vitro assays confirmed favorable biocompatibility and efficient uptake by 4T1 breast cancer cells without inducing adverse effects. The time-dependent in vivo NIR-II FL imaging demonstrated robust tumor accumulation of DSNPs, with maximum FL signals observed at 3 h post-injection, indicating effective passive tumor targeting via the EPR effect. The T1-weighted MRI also exhibited strong contrast enhancement at corresponding time points and demonstrated dual-modal diagnostic capability. The high signal-to-background ratio in both modalities enabled clear tumor delineation and facilitated precise NIR-II FL imaging-guided surgical resection with real-time visualization of tumor margins. Postoperative histological studies and blood biochemistry confirmed negligible acute systemic toxicity and supported the favorable in vivo biosafety of the F-127 polymer-coated DSNPs. In summary, the rationally designed DSNPs integrate high-sensitivity NIR-II FL imaging, effective T1-weighted MRI contrast, and excellent biocompatibility in a single nanoplatform. They provide a robust and clinically relevant tool for deep-tissue multimodal imaging and FL imaging-guided surgery. These findings highlight the translational potential of the dopant-engineered DSNPs for precision oncology.

## 2. Results and Discussion

### 2.1. Physicochemical Characterizations of the Prepared DSNPs

In this work, we deliberately designed dopant-engineered DSNPs by drawing on established principles of lanthanide energy transfer and MRI contrast generation for integrating NIR-II FL and T1-weighted MR imaging into a single nanoplatform. An optically transparent NaGdF_4_ host lattice was chosen because its low phonon energy effectively suppresses non-radiative losses and allows for efficient NIR-II emission [31]. The embedded Gd^3+^ ions in the host lattice simultaneously provided strong T1 contrast through enhanced proton relaxation. Nd^3+^ was incorporated as the primary sensitizer to enable excitation at 808 nm, which is more tissue-friendly than 980 nm excitation and reduces the thermal effect in vivo. Yb^3+^ acted as an efficient energy relay between Nd^3+^ and Er^3+^, while Er^3+^ served as the NIR-II emitter, producing characteristic emissions around 1334 nm and 1521 nm [17]. By systematically varying the Nd^3+^ content (2.5–7.5%) while keeping the Yb^3+^ and Er^3+^ concentrations constant, we were able to engineer the balance between light absorption, energy transfer efficiency, and the concentration-dependent quenching effect. This rational dopant engineering approach allowed us to simultaneously enhance NIR-II FL output and maintain high T1 relaxivity [32]. This strategy provided a solid and intuitive design basis for multimodal imaging and in vivo imaging-guided surgical studies.

**Figure 1:**
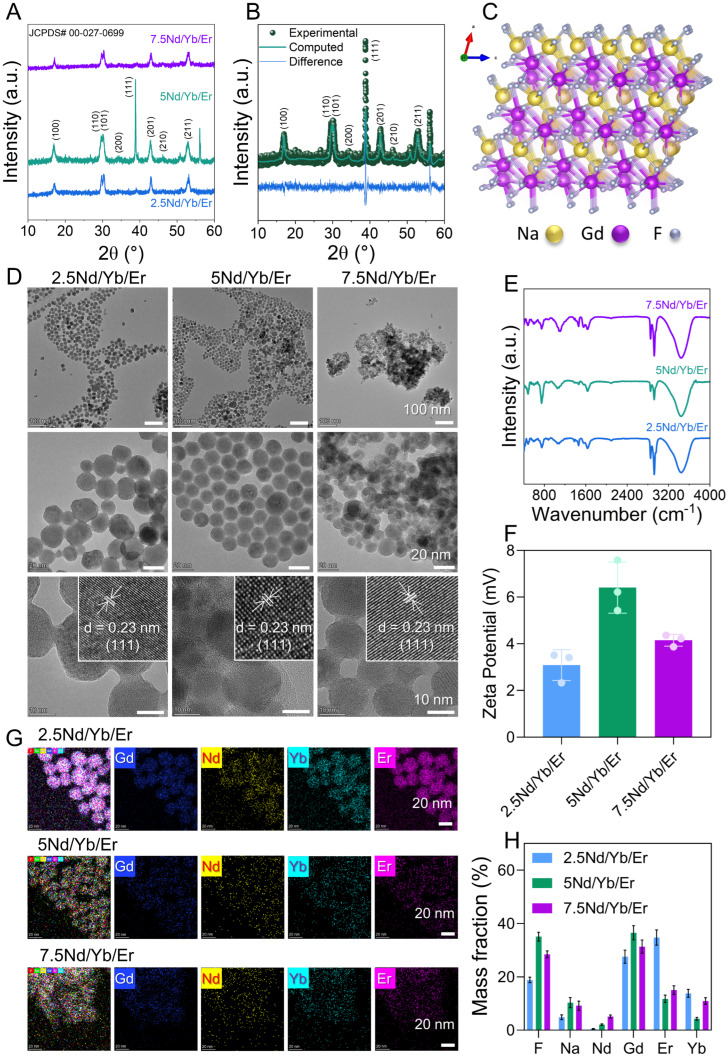
(**A**) The XRD spectra of the 2.5Nd/Yb/Er, 5Nd/Yb/Er, and 7.5Nd/Yb/Er DSNPs represent the phase purity and crystallinity. (**B**,**C**) Rietveld refinement of the XRD spectra and the derived crystalline structure of the 5Nd/Yb/Er DSNPs. (**D**) TEM images of the 2.5Nd/Yb/Er, 5Nd/Yb/Er, and 7.5Nd/Yb/Er DSNPs demonstrate the morphology and size of the prepared DSNPs (top row: scale bars = 100 nm; middle row: scale bars = 20 nm; and bottom row: scale bars = 10 nm). (**E**) FTIR spectra of the 2.5Nd/Yb/Er, 5Nd/Yb/Er, and 7.5Nd/Yb/Er DSNPs represent bond vibrations and atomic network formation. (**F**) Zeta potential of the 2.5Nd/Yb/Er, 5Nd/Yb/Er, and 7.5Nd/Yb/Er DSNPs demonstrates the surface charge density at pH 7.4. (**G**) EDS elemental mapping of the 2.5Nd/Yb/Er, 5Nd/Yb/Er, and 7.5Nd/Yb/Er DSNPs demonstrates the inclusion of all the component elements (F, Na, Nd, Gd, Er, and Yb; scale bars = 20 nm). (**H**) Mass fraction (%) of the component elements (F, Na, Nd, Gd, Er, and Yb) in the 2.5Nd/Yb/Er, 5Nd/Yb/Er, and 7.5Nd/Yb/Er DSNPs.

The crystallinity and phase purity of the synthesized DSNPs were extensively evaluated by X-ray diffraction (XRD) analysis to confirm their successful synthesis. The XRD spectra revealed a series of sharp and well-defined diffraction peaks in the (100), (110), (101), (111), (201), and (211) planes that match closely with the hexagonal β-phase NaGdF_4_ (JCPDS No. 00-027-0699), confirming the formation of the desired crystalline structure (Figure 1A). The absence of secondary phases and the sharpness of the peaks indicate excellent phase purity and successful incorporation of Nd^3+^, Yb^3+^, and Er^3+^ ions in the prepared DSNPs. No peak shifting with increasing Nd^3+^ concentration suggests that the dopant ions substitute the Gd^3+^ sites in an isostructural manner without inducing significant lattice distortion because of their comparable ionic radii [32]. Rietveld refinement was performed by using 5Nd/Yb/Er DSNPs as a representative sample to further confirm the crystal structure (Figure 1B) [33]. The refined patterns showed excellent agreement between the experimental and calculated values with minimal residuals. This confirms the high crystallinity and structural integrity of the hexagonal β-phase NaGdF_4_ lattice. The derived crystal microstructure demonstrated a fluoride-rich coordination environment (Figure 1C). This kind of coordination is well known to suppress non-radiative relaxation by minimizing phonon-assisted energy loss and could be an essential factor for achieving efficient NIR-II FL.

Transmission electron microscopy (TEM) was employed to investigate the morphology and size distribution of the DSNPs (Figure 1D). The 2.5Nd/Yb/Er DSNPs exhibit uniform spherical morphology with an average size of 15–20 nm. Increasing the Nd^3+^ concentration up to 5% in 5Nd/Yb/Er shows highly monodispersed DSNPs with well-defined lattice fringes in a similar size range. The measured interplanar spacing of 0.23 nm corresponds to the (111) plane of hexagonal β-phase NaGdF_4_ [21]. In contrast, the 7.5Nd/Yb/Er DSNPs show noticeable aggregation and reduced morphological uniformity. This indicates that excessive Nd^3+^ doping may induce lattice strain and defect formation, thereby affecting nucleation and growth behavior. These observations suggest that moderate Nd^3+^ doping is critical for maintaining optimal crystallinity and dispersibility. After that, Fourier transform infrared (FTIR) spectroscopy was employed to investigate the bonding environment of the DSNPs with different Nd^3+^ doping levels (Figure 1E). All variants of the DSNPs (2.5Nd/Yb/Er, 5Nd/Yb/Er, and 7.5Nd/Yb/Er) exhibit highly similar FTIR spectra. This indicates that the variation in Nd^3+^ content does not significantly alter the chemical structure of the DSNPs. The broad absorption band around ~3400 cm^−1^ was observed in all DSNPs variants and can be attributed to the stretching vibration of hydroxyl (-OH) groups [34]. Additionally, a weaker band near ~1630 cm^−1^ may correspond to the bending vibrations of H-O-H. This further confirms the presence of physically adsorbed water on the surface of DSNPs. The absence of distinct metal–oxygen vibration bands also confirms that the DSNPs remain in a fluoride coordination environment and do not form oxide or oxyfluoride phases.

The colloidal stability and surface charge density of the DSNPs were evaluated by zeta-potential measurements (Figure 1F). All the DSNPs variants (2.5Nd/Yb/Er, 5Nd/Yb/Er, and 7.5Nd/Yb/Er) exhibit positive surface potentials of 3.08 mV, 6.40 mV, and 4.14 mV, respectively, at pH 7.4. This can be attributed to the exposed lanthanide ions on the surface of DSNPs, which contribute to the formation of a positively biased electrical double layer in aqueous media. Although F-127 is a non-ionic polymer, its adsorption onto the DSNPs surface can modify the interfacial environment and shift the shear plane during electrophoretic measurements, thereby influencing the measured zeta potential without introducing intrinsic charge. The 5Nd/Yb/Er DSNPs showed the highest surface charge among all the variants [35]. This suggests that 5Nd/Yb/Er DSNPs possess superior dispersion stability in aqueous media (Figure S4). The reduction in surface charge density observed in 7.5Nd/Yb/Er DSNPs can be attributed to the Nd^3+^-induced aggregation according to the TEM results.

**Figure 2:**
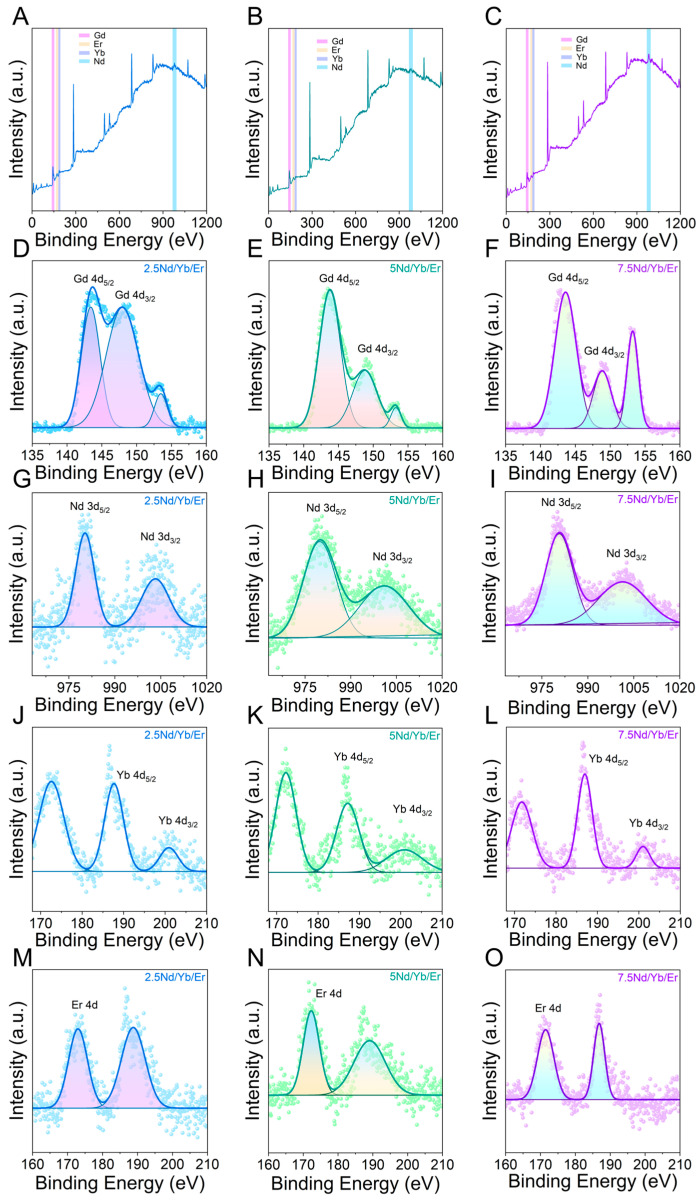
(**A**–**C**) The XPS survey for the 2.5Nd/Yb/Er, 5Nd/Yb/Er, and 7.5Nd/Yb/Er DSNPs demonstrates the elemental composition and valence state of the component elements. High-resolution XPS spectra demonstrate the presence of (**D**–**F**) Gd 4d, (**G**–**I**) Nd 3d, (**J**–**L**) Yb 4d, and (**M**–**O**) Er 4d in the 2.5Nd/Yb/Er, 5Nd/Yb/Er, and 7.5Nd/Yb/Er DSNPs, respectively.

Elemental composition and dopant distribution were analyzed by energy-dispersive X-ray spectroscopy (EDS) mapping (Figure 1G and Figure S1). A uniform spatial distribution of Na, Gd, F, Nd, Yb, and Er was observed for all DSNPs (2.5Nd/Yb/Er, 5Nd/Yb/Er, and 7.5Nd/Yb/Er). This confirms the homogenous incorporation of all constituent elements without phase segregation. The measured mass fractions confirm a progressive increase in Nd content with varying doping levels, while Gd content exhibits only minor fluctuations without a strictly monotonic decrease (Figure 1H) [1]. These results nevertheless demonstrate effective compositional tuning and controlled incorporation of Nd during synthesis. This kind of homogenous multiple-dopant engineering is essential for efficient energy transfer processes and reliable dual-modal imaging. X-ray photoelectron spectroscopy (XPS) was employed to investigate the elemental composition and chemical states of the component elements in DSNPs. The survey spectra of the 2.5Nd/Yb/Er, 5Nd/Yb/Er, and 7.5Nd/Yb/Er DSNPs clearly reveal the presence of Na, Gd, F, Nd, Yb, and Er in the DSNPs (Figure 2A–C). This confirms the successful incorporation of all constituent elements without detectable impurities. The gradual increase in the relative intensity of Nd- related signals with increasing Nd^3+^ content further verifies precise compositional control. High-resolution XPS spectra provide deeper insight into the chemical states of the individual lanthanide ions. The Gd 4d spectra display well-defined spin–orbit doublets corresponding to Gd 4d_5/2_ and Gd 4d_3/2_ at 143.5 eV and 148.7 eV binding energies, respectively (Figure 2D–F) [36]. The spectra also show binding energies characteristic of Gd^3+^ coordinated by fluoride ions. Notably, no discernible peak shifts or additional components are observed as the Nd^3+^ concentration increases. This indicates that Gd^3+^ maintains a stable oxidation state and local coordination environment despite partial substitution by dopant ions.

The Nd 3d spectra exhibit two prominent peaks assigned to Nd 3d_5/2_ and Nd 3d_3/2_ at 980.7 eV and 1001.3 eV, respectively (Figure 2G–I). This confirms the presence of Nd in the trivalent state. The systematic enhancement of the Nd peak intensity from the 2.5Nd/Yb/Er, 5Nd/Yb/Er, and 7.5Nd/Yb/Er DSNPs further corroborates the controlled incorporation of Nd^3+^ ions into the host lattice. Similarly, the Yb 4d at 186.9 eV (Figure 2J–L) and Er 4d at 172.9 eV (Figure 2M–O) in the high-resolution spectra confirm that both Yb and Er remain in the stable trivalent oxidation state across all variants. The nearly similar binding energies and spectral profiles observed for Yb^3+^ and Er^3+^ indicate that varying the Nd content does not perturb the chemical environments of these sensitizer and activator ions [37]. This kind of chemical stability is particularly important for maintaining efficient energy transfer pathways and can enhance the NIR-II FL performance of DSNPs. The structural integrity, homogenous lanthanide distribution, and stable trivalent states confirm that controlled Nd^3+^ doping preserves an efficient energy transfer network in DSNPs. Moderate Nd incorporation optimizes crystallinity and dispersion, providing the essential material basis for strong and reliable NIR-II FL suitable for imaging-guided surgical applications.

### 2.2. Investigation of NIR-II FL and MR Imaging Properties of DSNPs

**Figure 3:**
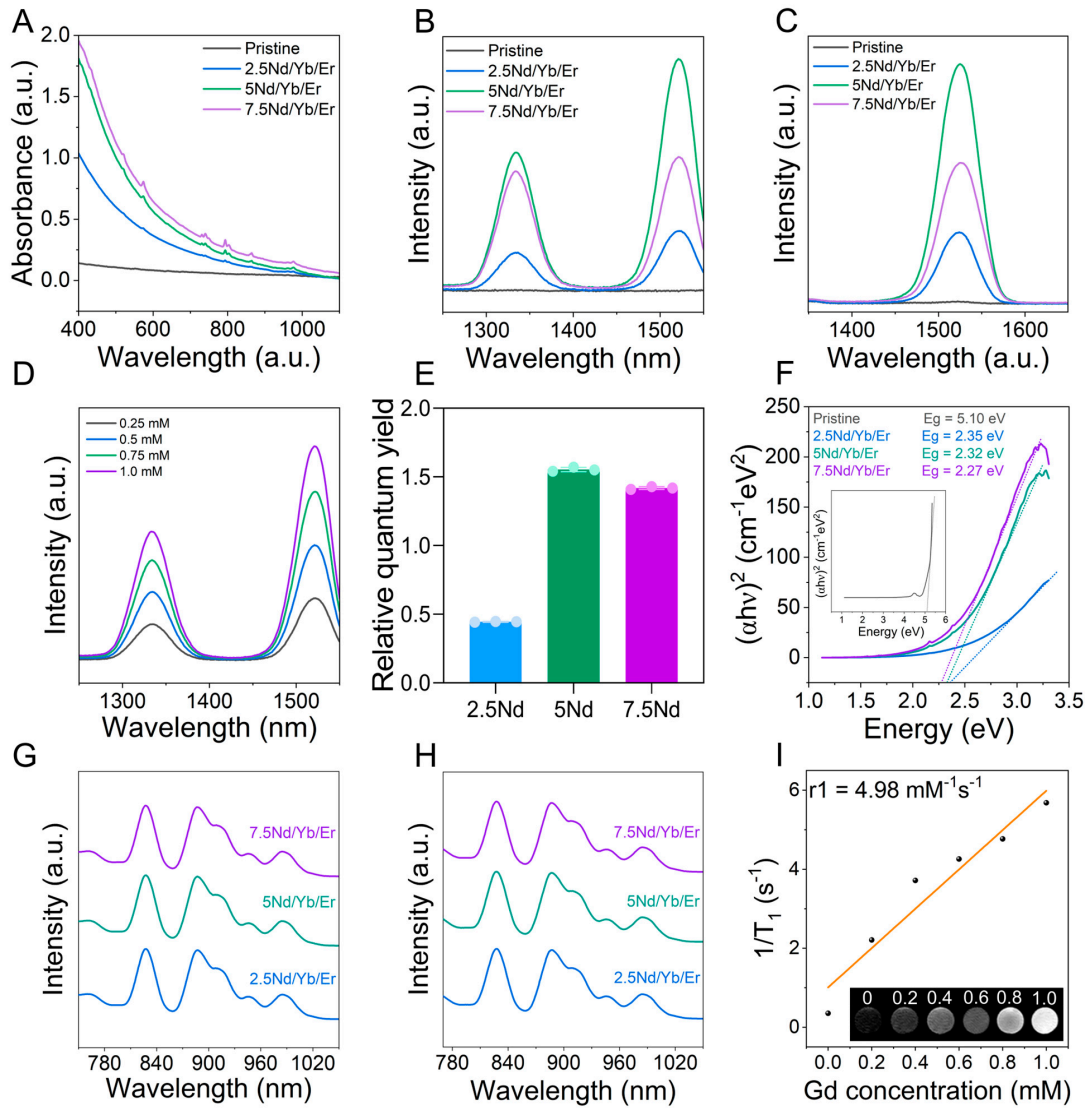
In vitro NIR-II FL and MR imaging capabilities of the 2.5Nd/Yb/Er, 5Nd/Yb/Er, and 7.5Nd/Yb/Er DSNPs. (**A**) The UV–Vis spectra of the 2.5Nd/Yb/Er, 5Nd/Yb/Er, and 7.5Nd/Yb/Er DSNPs represent the corresponding absorption peaks at 808 nm and 980 nm. The FL spectra of the 2.5Nd/Yb/Er, 5Nd/Yb/Er, and 7.5Nd/Yb/Er DSNPs represent the corresponding peaks at (**B**) 1334 nm and 1521 nm upon 808 nm laser excitation and at (**C**) 1521 nm upon 980 nm laser excitation. (**D**) The FL spectra of the 5Nd/Yb/Er DSNPs at different concentrations (0.25, 0.5, 0.75, and 1.0 mM) upon 808 nm laser excitation. (**E**) The relative Φ of the 2.5Nd/Yb/Er, 5Nd/Yb/Er, and 7.5Nd/Yb/Er DSNPs with respect to IR-783 dye. (**F**) The bandgap of the 2.5Nd/Yb/Er, 5Nd/Yb/Er, and 7.5Nd/Yb/Er DSNPs was measured via Tauc’s plot. The excitation spectra of the 2.5Nd/Yb/Er, 5Nd/Yb/Er, and 7.5Nd/Yb/Er DSNPs were measured at (**G**) 1334 nm and (**H**) 1521 nm excitations, respectively. (**I**) T1 relaxivity (r1) and the corresponding phantom images of the 5Nd/Yb/Er DSNPs at different concentrations (0, 0.2, 0.4, 0.6, 0.8, and 1.0 mM) of Gd.

The optical absorption properties of the DSNPs were investigated by using UV–Vis–IR spectroscopy (Figure 3A). All the DSNPs variants (2.5Nd/Yb/Er, 5Nd/Yb/Er, and 7.5Nd/Yb/Er) exhibit pronounced absorption bands at ~808 nm and ~980 nm as compared to the pristine NaGdF_4_ [17]. These absorption peaks correspond to the characteristic transitions of Nd^3+^ and Yb^3+^ ions. The absorption intensities gradually increase with increasing Nd^3+^ concentration. This confirms the role of Nd^3+^ as an efficient NIR sensitizer that can enable dual-wavelength excitation when co-doped with Yb^3+^ and Er^3+^ for NIR-II FL imaging. The DSNPs exhibit two strong NIR-II emission peaks at 1334 nm and 1521 nm upon 808 nm laser excitation (Figure 3B). This is because the NIR-II FL behavior of the DSNPs is governed by a multiple-step energy harvesting and transfer process that is highly sensitive to Nd^3+^ concentration. Under 808 nm excitation, Nd^3+^ ions are promoted to the ^4^F_3/2_ level and relax non-radiatively to lower sublevels, from which they exhibit characteristic emissions including a ^4^F_3/2_ → ^4^I_13/2_ band at ~1334 nm [38]. Moreover, the Nd^3+^ ions act as the primary sensitizers due to their strong absorption corresponding to the ^4^I_9/2_ → ^4^F_5/2_ or ^4^F_7/2_ transitions [39]. The excited Nd^3+^ ions rapidly relax non-radiatively to the metastable ^4^F_3/2_ level, and the energy is transferred to neighboring Yb^3+^ ions through resonant energy transfer. The Yb^3+^ ions subsequently serve as an energy relay and transfer the excitation energy to the Er^3+^ ion. This transfer populates the ^4^I_13/2_ level of Er^3+^ and results in intense NIR-II emissions at 1521 nm [40]. The 5Nd/Yb/Er DSNPs showed the highest emission intensity among all three compositions [41]. This dependence of emission intensity on Nd^3+^ concentration arises from a balance between energy harvesting efficiency and non-radiative loss mechanisms. The insufficient sensitizer density limits light absorption and reduces the probability of Nd → Yb energy transfer at low Nd^3+^ content (2.5%). The increase in Nd^3+^ concentration (5%) enhances excitation capture and promotes efficient interionic energy transfer. This phenomenon yields maximum NIR-II FL intensity. However, a further increase in Nd^3+^ content (7.5%) introduces Nd-Nd cross-relaxation pathways and energy back-transfer processes. It increases the non-radiative decay and reduces the overall emission efficiency, termed as concentration quenching.

On the other hand, only the 1521 nm emission peak is observed under 980 nm laser excitation (Figure 3C). In this case, the excitation selectively addresses Yb^3+^ ions that transfer energy directly to Er^3+^ without the involvement of Nd^3+^ and results in an emission at 1521 nm. However, the presence of Nd3+ can still influence the overall emission efficiency through lattice-mediated energy migration and possible interionic interactions. This excitation-dependent emission behavior confirms the distinct yet complementary roles of Nd^3+^ and Yb^3+^ as sensitizers within the DSNPs [42]. Moreover, the FL intensity of the 5Nd/Yb/Er DSNPs increased monotonically with the concentration (Figure 3D). This increment demonstrates stable emission behavior without aggregation-induced quenching.

To further validate the advantage of optimized dopant-engineering of Nd^3+^, the relative quantum yield (Φ) of all the DSNPs variants was calculated by using IR-783 dye as a reference (Figure S2). The quantitative analysis revealed that the 5Nd/Yb/Er DSNPs possess the highest relative Φ (1.55) among all the other variants (Figure 3E) [43]. The Tauc plot analysis shows that lanthanide doping significantly narrows the bandgap of NaGdF_4_ from 5.10 eV to 2.35 eV, 2.32 eV, and 2.27 eV in the 2.5Nd/Yb/Er, 5Nd/Yb/Er, and 7.5Nd/Yb/Er DSNPs, respectively (Figure 3F). The reduction in optical bandgap upon lanthanide doping further contributes to enhanced NIR absorption by introducing localized 4f energy states within the host lattice [44]. These states facilitate efficient photon capture and suppress non-radiative relaxation by leveraging the low phonon energy environment of the fluoride lattice and preserving excited states for radiative transitions. The excitation spectra monitored at 1334 nm and 1521 nm further corroborate the efficient excitation of Nd^3+^ and Yb^3+^ ions and support the proposed multiple-step energy transfer mechanism.

The DSNPs also exhibit excellent MRI capability due to the presence of Gd^3+^ ions in the optically transparent NaGdF_4_ matrix. The T1 relaxivity of the 5Nd/Yb/Er DSNPs was determined to be 4.98 s^−1^ mM^−1^ and is comparable to that of clinically used Gd-based contrast agents (Figure 3I) [45,46]. The high T1 relaxivity arises from the abundant Gd^3+^ ions with seven unpaired 4f electrons. These unpaired electrons are responsible for strongly enhancing the longitudinal relaxation of protons through dipole–dipole interactions with surrounding water molecules. The preserved crystal structure and surface accessibility of Gd^3+^ ensure efficient water exchange dynamics. This results in linear and concentration-dependent T1 signal enhancement [47]. The corresponding phantom images display concentration-dependent signal enhancement and confirm the effectiveness of 5Nd/Yb/Er DSNPs as positive MRI contrast agents. Therefore, the optimized dopant-engineered 5Nd/Yb/Er DSNPs integrate strong NIR-II FL with high T1 relaxivity for dual-modal NIR-II FL and MR imaging. This synergistic optical and magnetic performance makes them highly suitable for high-contrast imaging and NIR-II FL imaging-guided surgical applications.

### 2.3. In Vitro Biocompatibility and Cellular Internalization of DSNPs

**Figure 4:**
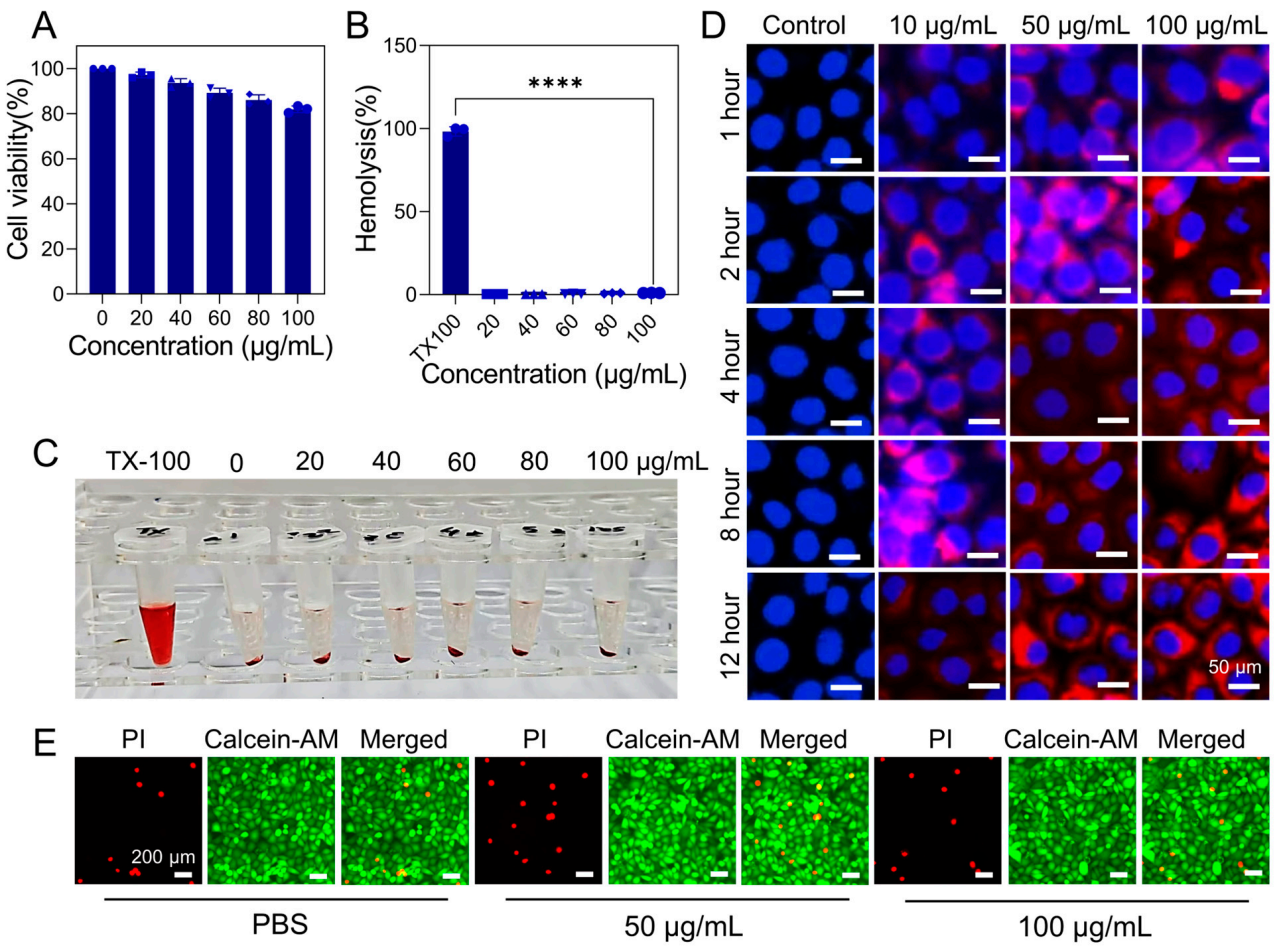
In vitro biocompatibility assessment of the 5Nd/Yb/Er DSNPs. (**A**) Cell viability assay of the 4T1 cell line at different concentrations (0, 20, 40, 60, 80, and 100 µg/mL) of the 5Nd/Yb/Er DSNPs. (**B**,**C**) Hemolysis assay and representative image of different concentrations (20, 40, 60, 80, and 100 µg/mL) of the 5Nd/Yb/Er DSNPs and TX100 (ordinary one-way ANOVA: **** *p* > 0.0001). (**D**) Cellular internalization of 5Nd/Yb/Er DSNPs in 4T1 cells at different concentrations (0, 10, 50, and 100 µg/mL) in 1–12 h time intervals (scale bars = 25 µm). (**E**) Live/dead assay of 4T1 cell line at different concentrations (0, 50, and 100 µg/mL) of 5Nd/Yb/Er DSNPs (scale bars = 200 µm).

The in vitro biocompatibility of the optimized 5Nd/Yb/Er DSNPs coated with Pluronic F-127 was systematically evaluated by using the 4T1 breast cancer cell line [48]. As shown in Figure 4A, the DSNPs exhibited negligible cytotoxicity as the cell viability remained above ~85% across the tested concentration range (0–100 µg/mL). This excellent biocompatibility can be attributed not only to the chemically stable fluoride host lattice but also to the presence of the F-127 polymer coating [49,50]. This polymer coating is particularly responsible for effectively shielding the inorganic core, reducing the direct interaction of DSNPs with the cellular environment, and improving the colloidal stability under physiological conditions [51]. The hemolysis assay further confirmed the blood compatibility of the F-127 polymer-coated DSNPs. As depicted in Figure 4B,C, the hemolysis percentage remained below 5% at all tested concentrations (0–100 µg/mL) of DSNPs as compared to the positive control (TX-100), which induced severe hemolysis. The amphiphilic structure of F-127 forms a hydrated steric barrier on the surface of DSNPs to prevent membrane disruption and non-specific protein adsorption, which is essential for safe intravenous administration [52].

The cellular internalization studies revealed a clear concentration and time-dependent increment in intracellular FL signals (Figure 4D). This efficient cellular internalization can be attributed to the biocompatible F-127 polymer coating. It is responsible for enhanced colloidal stability in the physiological environment and makes the DSNPs bioavailable for the tumor cells. The live/dead staining results further confirm excellent biocompatibility by showing predominantly Calcine-Am-positive cells (green) and minimal PI staining (red) even at 100 µg/mL (Figure 4E). Therefore, the biocompatibility results demonstrate that F-127 polymer-coated 5Nd/Yb/Er DSNPs are biologically safe with effective cellular uptake [53]. This establishes a solid biological basis for further in vivo NIR-II FL and MR imaging and NIR-II Fl imaging-guided surgical applications.

### 2.4. In Vivo NIR-II FL and MR Imaging and NIR-II FL Imaging-Guided Surgery

**Figure 5:**
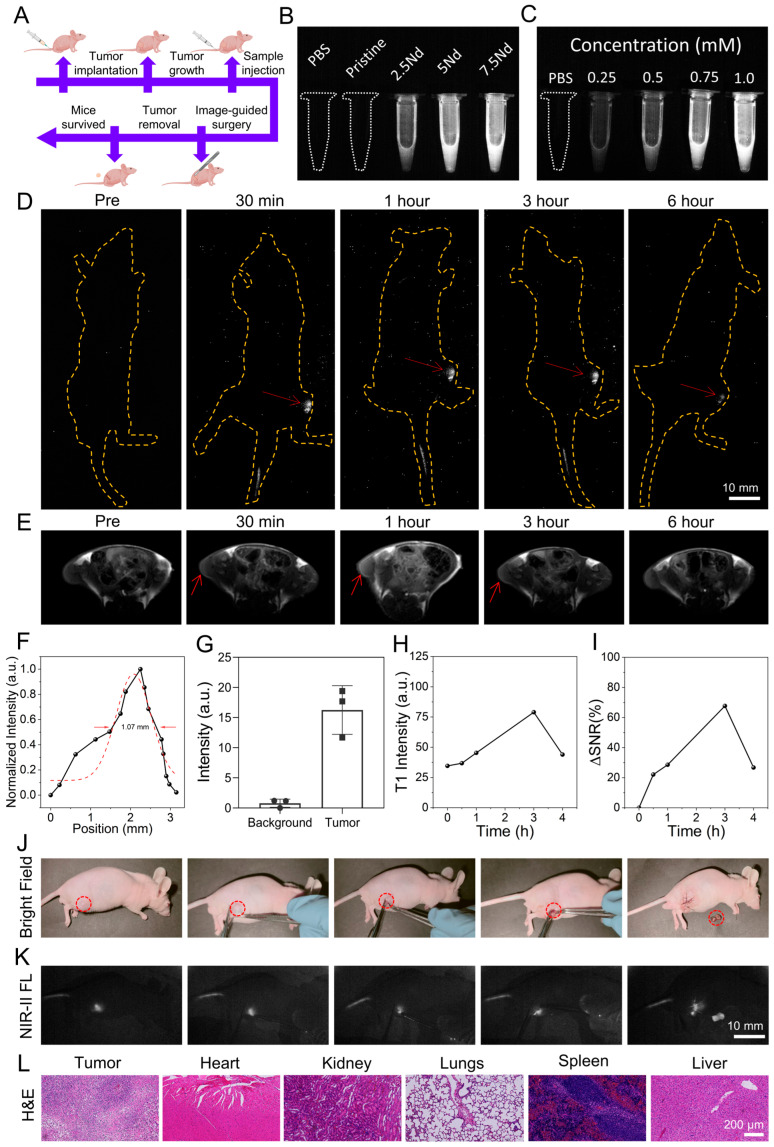
(**A**) Schematic illustration of the imaging-guided surgery of the subcutaneous 4T1 breast tumor-bearing mice. (**B**) In vitro NIR-II FL images of the 2.5Nd/Yb/Er, 5Nd/Yb/Er, and 7.5Nd/Yb/Er DSNPs. (**C**) In vitro NIR-II FL images at different concentrations (0, 0.25, 0.5, 0.75, and 1.0 mM) of the 5Nd/Yb/Er DSNPs. (**D**) In vivo NIR-II FL images of the subcutaneous 4T1 breast tumor-bearing mice at different time intervals post-injection (pre-injection, 30 min, 1 h, 3 h, and 6 h) (scale bar = 10 mm). (**E**) In vivo T1 phantom images of the subcutaneous 4T1 breast tumor-bearing mice at different time intervals post-injection (pre-injection, 30 min, 1 h, 3 h, and 6 h). (**F**) NIR-II FL intensity measured along the red dashed line, with the peak fitted to a Gaussian function for the 4T1 breast tumor-bearing mice imaging. (**G**) The comparison of NIR-II FL intensity within the tumor and the background. (**H**) T1 signal intensities and the corresponding (**I**) signal-to-noise ratio (SNR) for MR images at different time intervals (pre-injection, 30 min, 1 h, 3 h, and 6 h). (**J**) Bright field and (**K**) NIR-II FL imaging-guided surgery of the subcutaneous 4T1 breast tumor-bearing mice (scale bar = 10 mm). (**L**) H&E staining of the major body organs and 4T1 breast tumor after NIR-II FL imaging-guided surgery of the mice (scale bars = 200 µm). The red arrows in (**D**,**E**), and red circles in (**J**) represent tumor site.

The in vivo dual NIR-II FL and MR imaging performance and surgical applicability of the optimized F-127 polymer-coated 5Nd/Yb/Er DSNPs were systematically evaluated by employing subcutaneous 4T1 breast tumor-bearing mice (Figure 5A). As depicted in Figure 5B,C, the 5Nd/Yb/Er DSNPs exhibited the strongest in vitro NIR-II FL intensity among all compositions and demonstrated a significant concentration-dependent (0–1 mM) enhancement, which is consistent with the optimized Nd-Yb-Er energy transfer process [54,55]. The whole-body NIR-II FL imaging at different time intervals (0–6 h) demonstrated rapid tumor localization of the 5Nd/Yb/Er DSNPs as early as 30 min post-injection [56,57]. The in vivo FL intensity progressively increased and reached a maximum at 3 h post-injection (Figure 5D). This time-dependent accumulation indicates prolonged blood circulation and efficient tumor retention that can be attributed to the nanoscale size of the DSNPs and hydrophilic F-127 polymer coating. The F-127 polymer coating effectively reduces the non-specific protein adsorption and reticuloendothelial system uptake and also facilitates passive tumor targeting by the enhanced permeability and retention (EPR) effect [58]. Moreover, the quantitative NIR-II FL analysis further confirmed a pronounced signal contrast between the tumor and the surrounding tissues (Figure 5F,G). The FL intensity profile across the tumor region displayed a well-defined Gaussian distribution. This indicates homogeneous intratumoral distribution of the DSNPs and enables precise tumor boundary delineation. The high tumor-to-background ratio originates from the strong NIR-II emission at 1521 nm because of the substantially suppressed photon scattering and tissue autofluorescence [5,48]. In parallel, the T1-weighted MR imaging demonstrated a gradual enhancement of signal intensity at different time intervals (0–6 h) in the tumor site following the administration of DSNPs (Figure 5E). The MRI signals and the corresponding signal-to-noise ratio increased over time and peaked at approximately 3 h post-injection (Figure 5H,I). This is in close agreement with the NIR-II FL imaging results. This dual-modal correlation confirms the effective tumor accumulation of the Gd-containing DSNPs and highlights their application for high-resolution MR imaging in vivo. The observed MRI contrast enhancement is attributed to the high Gd content within the NaGdF_4_ host lattice and the favorable water accessibility enabled by the polymer-coated DSNPs.

Importantly, high-resolution NIR-II FL imaging was employed for the FL imaging-guided surgery of the subcutaneous 4T1 breast tumor-bearing mice. The NIR-II FL signals provided a clear real-time visualization of tumor boundaries during the surgery (Figure 5J,K). This imaging-guided surgery enabled precise identification and complete resection of tumor tissue and minimized the damage to surrounding healthy tissues [48,59]. The high spatial resolution and deep-tissue penetration associated with NIR-II emission allowed residual tumor regions to be readily distinguished from normal tissues (Figure S3). This reduces the major risk of incomplete excision in conventional surgery. The sustained FL signals during the operative window further indicate excellent photostability and in vivo signal retention of the DSNPs, which are critical for reliable intraoperative imaging guidance.

A postoperative histological examination (H&E staining) of the major body organs, including the heart, liver, spleen, lungs, and kidney, as well as tumor tissues, was conducted (Figure 5L). This revealed no noticeable inflammation, necrosis, or structural abnormalities. This confirms that the F-127 polymer-coated DSNPs do not induce acute organ toxicity or tissue damage under the applied imaging conditions. This favorable biosafety profile can be attributed to the stable fluoride-based host lattice, effective lanthanide ion confinement, and the biocompatible F-127 surface coating [60]. These collectively control the ion leakage and non-specific biological interactions. Therefore, these findings demonstrate that the rationally engineered 5Nd/Yb/Er DSNPs successfully integrate high-resolution NIR-II FL imaging with efficient MRI contrast enhancement and also maintain excellent in vivo biocompatibility. This dual-modal imaging capability enables accurate preoperative localization, real-time intraoperative guidance, and safe postoperative outcomes. It highlights the strong potential of the DSNPs for precision imaging-guided surgical interventions in breast cancer.

### 2.5. In Vivo Biosafety Evaluation by Blood Biochemistry Analysis

**Figure 6:**
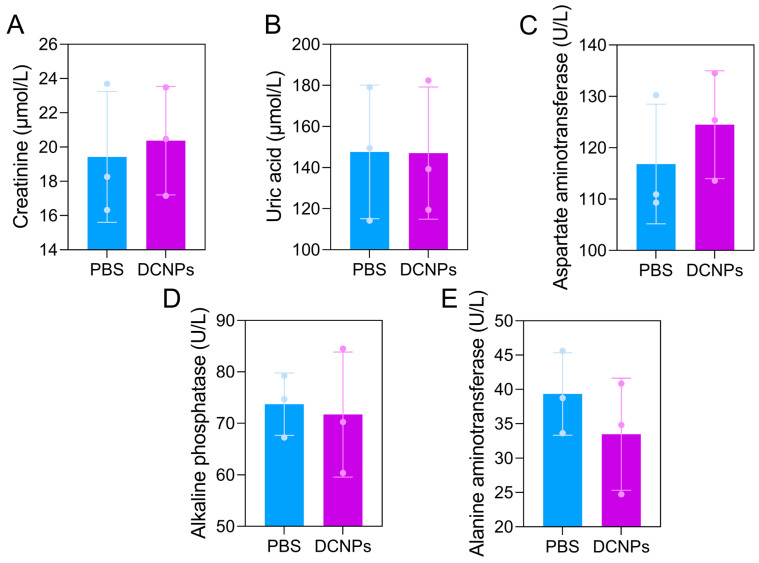
Blood biochemistry after the NIR-II FL imaging-guided surgery (n = 3). Mouse blood tests were conducted to estimate the (**A**) blood creatinine (Crea), (**B**) uric acid (UA), (**C**) aspartate aminotransferase (AST), (**D**) alkaline phosphatase (ALP), and (**E**) alanine aminotransferase (ALT) levels for evaluating the biosafety and biocompatibility of the 5Nd/Yb/Er DSNPs.

Comprehensive blood biochemistry analyses were performed for further assessment of the systemic biosafety of the 5Nd/Yb/Er DSNPs following the NIR-II FL imaging-guided surgery. Key renal function indicators, including blood creatinine (Crea) and uric acid (UA), showed no significant statistical differences between the DCNP-treated group and the control (PBS) group (Figure 6A,B). These results indicate that the administration of DSNPs does not impair glomerular filtration or renal excretory functions. Moreover, liver function was evaluated by measuring serum levels of aspartate aminotransferase (AST), alanine aminotransferase (ALT), and alkaline phosphatase (ALP) (Figure 6C–E) [61]. These are sensitive markers for hepatocellular injury and biliary dysfunction. The levels of AST, ALT, and ALP in the DCNP-treated mice remained within the normal physiological ranges and were comparable to those observed in the PBS group. The absence of enzyme elevation indicates that the DSNPs do not induce hepatocellular damage or inflammatory stress following systemic administration and surgical intervention.

This favorable blood biochemistry profile can be attributed to the intrinsic chemical stability of the NaGdF_4_ host lattice. It effectively confines lanthanide ions and minimizes ion leakage in vivo. Additionally, the F-127 polymer coating plays a critical role in enhancing colloidal stability, reducing non-specific protein adsorption, and controlling interactions with biological membranes. This phenomenon alleviates the potential hepatic and renal burden. These results demonstrate that the 5Nd/Yb/Er DSNPs exhibit excellent in vivo biocompatibility and systemic safety after imaging-guided surgery. This further supports their suitability for dual-modal NIR-II FL and MR imaging-assisted clinical translation.

## 3. Conclusions

In summary, the presented work demonstrated that precise dopant engineering offers an efficient route to unify high-resolution NIR-II FL and MR imaging within a single DCNP nanoplatform. By carefully adjusting the Nd^3+^ content in the optically transparent NaGdF_4_ matrix while maintaining fixed Yb^3+^ and Er^3+^ concentrations, the intrinsic link between energy transfer efficiency, emission intensity, and imaging performance was evaluated. This systematic approach identified the 5Nd/Yb/Er composition as an optimal balance point for efficient excitation harvesting and cascade energy transfer without detrimental concentration quenching. Besides optical optimization, the presence of Gd^3+^ in the optically transparent fluoride lattice enabled reliable T1-weighted MRI contrast, allowing for seamless integration of preoperative anatomical imaging with real-time intraoperative NIR-II FL guidance. Importantly, surface modification with Pluronic F-127 translated these physicochemical advantages into robust biological performance, supporting favorable biodistribution, biosafety, and a prolonged imaging window suitable for surgical interventions. Looking forward, this work provided a multifunctional lanthanide-based nanoplatform that bridges diagnosis and surgery. The insights presented in this work are broadly applicable for the development of next-generation dual-imaging agents and offer a practical foundation for advancing precision imaging and imaging-guided therapy in solid tumors.

## Data Availability

The original contributions presented in this study are included in the article/Supplementary Material. Further inquiries can be directed to the corresponding authors.

## Supplementary Materials

The following supporting information can be downloaded at: https://www.mdpi.com/article/10.3390/bios16030180/s1. References [4,7,13,17,20,23,25,26,46,48,49,50,51,52,55,56,57,58,59,62] are cited in the Supplementary Materials.
