# Supplementary material for: Dopant-Engineered Downshifting Nanoparticles with Dual NIR-II Fluorescence and Magnetic Resonance Imaging for Diagnosis and Image-Guided Surgery of Breast Cancer
Source: Biosensors (Basel). 2026 Mar 23;16(3):180. doi: 10.3390/bios16030180 (PMC13024726; doi:10.3390/bios16030180)
Supplement: Supplementary file 1 [file biosensors-16-00180-s001.zip › biosensors-4137515-supplementary.pdf]

Supplementary Information

Dopant-Engineered Downshifting Nanoparticles with Dual NIR-II Fluorescence and Magnetic Resonance Imaging for Diagnosis and Image-Guided Surgery of Breast Cancer

Zia Ullah [1,†], Mu Du [2,†], Lihong Jiang [3], Yibin Yan [1], Yuqian Yan [1], Jingsi Gu [3], Jing Cheng [3], Bing Guo [1,*] and Zun Wang [4,*]

[1] School of Science, Harbin Institute of Technology, Shenzhen 518055, China; 23sf58006@stu.hit.edu.cn (Z.U.); 25s058070@stu.hit.edu.cn (Y.Y.); 24s058082@stu.hit.edu.cn (Y.Y.)

[2] Department of Radiology, Southern University of Science and Technology Hospital, Shenzhen 518055, China; dudumumu@163.com

[3] Education Center of Experiments and Innovations, Harbin Institute of Technology, Shenzhen 518055, China; jianglihong@hit.edu.cn (L.J.); gujingsi@hit.edu.cn (J.G.); chengjing88@hit.edu.cn (J.C.)

[4] Department of Breast and Thyroid Surgery, Shenzhen Baoan Women's and Children's Hospital, Jinan University, Shenzhen 518000, China

* Correspondence: guobing2020@hit.edu.cn (B.G.); wzttkl@163.com (Z.W.)
† These authors contributed equally to this work.

1. Experimental Section

1.1. Materials

The chemicals used in the study include Gadolinium (III) acetate hydrate ($C_6H_9GdO_6.xH_2O$, 99%) Neodymium (III) acetate hydrate ($Nd(CH_3COO)_3.xH_2O$, 99.95%), Ytterbium (III) acetate hydrate ($Yb(C_2H_3O_2)_3.xH_2O$, 99.95%), Erbium (III) acetate hydrate ($Er(CH_3CO_2)_3.xH_2O$, 99.9%), Oleic acid (>99%), 1-octadecane (99%), Sodium hydroxide (Reagent grade), Ammonium fluoride (Reagent grade), Methanol (Analytical grade), Ethanol (Analytical grade), Cyclohexane (ACS grade), and Pluronic F-127. All these chemicals were sourced from Sigma-Aldrich, Macklin, and Aladdin. All the chemicals were used as received without further purification. The water used throughout all our experiments was de-ionized water having a resistivity of at least 18.2 MΩ.cm.

1.2. Synthesis of the NIR-II fluorescent downconversion nanoparticles (DCNPs)

1.2.1. Synthesis of $NaGd_{0.7-x}F_4$@$Nd_xYb_{0.25}Er_{0.05}$ DCNPs

$C_6H_9GdO_6.xH_2O$ (0.65 mmol), $Nd(CH_3COO)_3.xH_2O$ (0.05 mmol), $Yb(C_2H_3O_2)_3{\cdot}xH_2O$ (0.25 mmol), and $Er(CH_3CO_2)_3{\cdot}xH_2O$ (0.05 mmol) were added into a three-neck flask containing 9 ml of oleic acid and 22.5ml of 1-octadecane, and the reaction mixture was stirred for 15 min. Then the temperature was increased to 150 °C after closing the rubber caps to avoid oxidation for 20-30 min. The reaction mixture was then cooled to 50 °C, and a mixture of NaOH (2.5 mmol) and NH_4F (4 mmol) in 20 mL of methanol was added dropwise, continued stirring for 45 min at 50 °C, and then the temperature was raised to 75°C to evaporate methanol. The three-neck flask was sealed again and kept in a vacuum condition for 10 to 15 minutes. The solution temperature was increased to 100 °C for 10 min to remove the traces of methanol. The mixture was heated to 300 °C under an argon atmosphere with vigorous stirring at ~600 RPM for 1 hour. The solution was cooled to room temperature, and the absolute ethanol was added to the solution in the same volume for participation. The whole mixture was centrifuged and washed several times. Finally, the nanoparticles designated as 5Nd/Yb/Er DCNPs were dispersed in 5 mL cyclohexane and kept at 4°C for further usage.

The other variants designated as 2.5Nd/Yb/Er and 7.5Nd/Yb/Er DCNPs were prepared by adding 0.025 and 0.075 mmol of $Nd(CH_3COO)_3.xH_2O$, respectively, following a similar procedure.

1.2.2. Functionalization of the NIR-II fluorescent DCNPs

For water dispersibility, the DCNPs were functionalized with a chloride group. For that, 30 mg of DCNPs were dispersed in 15 mL of ethanol, and then 115 µL conc. HCl was added. The resulting mixture was sonicated for 30 min and stirred for another 30 min to uniformly functionalize the DCNPs. The chloride-functionalized DCNPs were centrifuged and washed with acidic ethanol (pH 4) once and with neutral ethanol twice. Finally, the functionalized DCNPs were washed with DI water, dried, and stored for further characterization.

1.2.3. Pluronic F-127 coating of the functionalized DCNPs

For biocompatibility, the functionalized DCNPs were further coated with Pluronic F-127 polymer. For that, 100 mg of the Pluronic F-127 was added to 20 mL of DI water and

stirred until the formation of a clear solution. After that, 100 mg of the functionalized DCNPs were added to the solution and stirred for at least 12 hours for uniform coating. The F-127-coated DCNPs were then washed thrice to remove the residual polymer and stored for further experiments.

1.3. Characterizations of the NIR-II fluorescent DCNPs

The crystallinity and phase purity analysis of DCNPs was conducted using an X-ray powder diffractometer (XRD, AREIS, PANalytical). The study was performed across a 2θ range of 10° to 80°, using a diffractometer set to 35 kV and 35 mA [1]. A Cu-Kα radiation source with a wavelength of 1.5418 Å was used, operating at a scan speed of 0.5 sec/step [2]. The Rietveld refinement technique was employed to obtain information about the microstructure and crystal parameters [3]. The measured diffractograms were refined by employing MAUD v2.996 software. The refined microstructures and their bonding networks were represented by employing the VESTA v3.5.2 software. Transmission Electron Microscopy (TEM, Hitachi HT7800) was conducted to investigate the morphology of the samples under the accelerating voltage of 120 kV. After being dispersed in chloroform, the samples were applied dropwise onto a 300-mesh copper grid and then dried in a vacuum [2].

The phases and vibrational modes of the synthesized samples were analyzed using a Thermo Scientific SMART iTR Fourier transform infrared (FTIR) spectrometer [2]. The measurements were conducted across a wavenumber range of 400 to 4000 cm^{-1}. The surface potential of the samples was measured on a Malvern Zetasizer instrument (ZEN3690, Malvern PANalytical Limited, U.K.) [4]. After that, the energy dispersive X-ray spectroscopy (EDS) spectra and elemental mapping were taken using a Thermo Fly TF-G20 TEM with an operating voltage of 120 kV. The Thermo Scientific K-ALPHA instrument was used for the X-ray photoelectron spectroscopy (XPS) of the DCNPs by using a Monochromatic Al target (E = 1486.68 eV) source for determining the chemical states of the component elements present [5].

1.4. Optical properties of the NIR-II fluorescent DCNPs

The optical absorbance spectra of the DCNPs were measured by employing a UV-visible spectrophotometer (UV579, Hunan Qianyan Technology Co., Ltd., China), ranging from 200 nm to 1100 nm, to evaluate the absorption spectra of the DCNPs [2]. After that,

the fluorescence spectra of the DCNPs were measured by employing a spectrofluorometer (Fluoromax-Plus, HORIBA Scientific, USA). The DCNPs were excited by 808 nm and 980 nm lasers, and the fluorescence spectra were obtained ranging from 1100 nm to 1600 nm. Moreover, the excitation spectra (λ_{ex}) of the DCNPs were also measured by employing the same spectrofluorometer [2,6]. The emission monochromators were fixed at the emission maxima (λ_{em} = 1334 nm and 1521 nm), and excitation was scanned from 700 nm to 1100 nm. The resulting λ_{ex} was then normalized to unity at the maximum to facilitate comparison with the UV-visible absorption profile of DCNPs [2,7].

The NIR-II fluorescence quantum yield (Φ) of the DCNPs was evaluated using a relative method, with IR-783 dye (Φ = 0.11) serving as the reference standard due to its well-matched absorption and emission properties in the NIR region. Serial dilutions of the DCNPs and IR-783 dye solutions were prepared, maintaining the absorbance at the 808 nm excitation wavelength below 0.1 to minimize inner-filter and reabsorption effects [8]. Absorption spectra were recorded using a UV-visible spectrophotometer, and fluorescence emission spectra were acquired with a spectrofluorometer under identical measurement conditions. Both samples were excited at 808 nm, and emission spectra were collected over the 1100-1600 nm range [9]. The emission spectra were integrated to obtain the total fluorescence intensity for each dilution, and the resulting integrated intensities were plotted against the corresponding absorbance value at the excitation wavelength to construct linear calibration curves for the DCNPs and IR-783dye. The slopes of the corresponding curves ($Slope_{DCNPs}$ and $Slope_{IR-783}$) were calculated using linear regression analysis [10]. The relative Φ of the DCNPs was then calculated using the mentioned equation:

$$\Phi_{DCNCs} = \Phi_{IR-783} \frac{Slope_{DCNCs}}{Slope_{IR-783}} \left(\frac{n_{DCNCs}}{n_{IR-783}}\right)^2 \quad \text{(S1)}$$

Here, $Slope_{DCNPs}$ and $Slope_{IR-783}$ are the slopes of the emission *vs* absorbance plots for DCNPs and IR-783. The n_{DCNPs} and n_{IR-783} are the refractive indices of the solvents used for the DCNPs and IR-783 dye [9].

1.5. *In vitro* NIR-II fluorescence studies

The *in vitro* fluorescence images of the DCNPs were captured in a Teledyne NIR-II fluorescence imaging system (Princeton Instruments, USA. Ex, 808 nm; long-pass filter, 1000 nm; exposure time, 1000 ms; power density, 40 mW/cm^2). The acquired images were then processed and analyzed using the ImageJ software [11].

1.6. *In vitro* MRI studies

MRI measurements were performed *in vitro* to evaluate the longitudinal relaxivity (r1) of the DCNPs [12]. A series of aqueous solutions of the sample with different Gd^{3+} concentrations (0 to 1 mM) were prepared in 500 μL microtubes, using distilled water as the diamagnetic control. The T_1-weighted phantom images and quantitative T_1 mapping of DCNPs were acquired on a Philips Elition 3.0 Tesla clinical MRI system at 30 °C. The longitudinal relaxation times (T_1) were extracted by fitting the signal intensities to a mono-exponential recovery model. The relaxation rates ($1/T_1$) were plotted as a function of Gd^{3+} concentration, and the longitudinal relaxivity (r1) was determined from the slope of the linear fit according to the relation:

$$\frac{1}{T_1} = \frac{1}{T_{1,0}} + r_1[Gd] \qquad \text{(S2)}$$

Here $\frac{1}{T_{1,0}}$ represents the relaxation rate of distilled water measured under identical conditions. The images were analyzed by employing RadiAntViewer and ImageJ software [13].

1.7. *In vivo* NIR-II fluorescence imaging of subcutaneous breast tumor-bearing mice

Female BALB/c mice (n = 5) were subjected to NIR-II fluorescence imaging *in vivo*. After 10 days of tumor inoculation, *in vivo* NIR-II fluorescence images were captured before and after the intravenous administration of 5Nd/Yb/Er DCNPs solution (10 mg/kg body weight) at different time intervals post-injection (pre, 30 minutes, 1 hour, 3 hours, and 6 hours) by employing Teledyne NIR-II fluorescence imaging system (Princeton Instruments, USA. Ex, 808 nm; long-pass filter, 1000 nm; exposure time, 1000 ms; power density, 40 mW/cm^2). The acquired images were then processed and analyzed by using the ImageJ software [13,14].

1.8. *In vivo* MR imaging of the subcutaneous breast tumor-bearing mice

Female BALB/c mice (n = 5) were subjected to MRI scans *in vivo*. After 10 days of tumor inoculation, *in vivo* MRI was conducted before and after the intravenous administration of 5Nd/Yb/Er DCNPs (10 mg/kg body weight). The T1WI phantom images of the subcutaneous 4T1 breast tumor-bearing mice were captured by employing a Philips Elition 3.0T MRI system at different time intervals (pre, 30 minutes, 1 hour, 3 hours, 4

hours, and 6 hours). The T1WI MR images were analyzed by using RadiAntViewer and ImageJ software [15].

1.9. Bio-experiments NIR-II fluorescence imaging-guided surgery of breast cancer

1.9.1. Cell viability assay of 5Nd/Yb/Er DCNPs

The cell viability assay was conducted to assess the cell viability of DCNPs. 4T1 breast cancer cells were seeded into 96-well plates at a density of 5 x 10^3 cells per well. After a 24-hour attachment period, the cells were incubated with different concentrations (0 to 100 µg/mL) of 5Nd/Yb/Er DCNPs for another 24 hours. Following incubation, the culture medium was replaced with 100 µL of fresh medium containing 10 µL of MTT solution (Solarbio, China). The plates were then incubated at 37 °C for 4 hours. After removing the medium, 100 µL of DMSO was added. Finally, the absorbance at 450 nm was measured using a BioTek Epoch microplate reader. The background absorbance from the empty wells was subtracted. Cell viability was calculated using the following equation:

$$Cell\ viability\ (\%) = \frac{A_{sample} - A_0}{A_{control} - A_0} \times 100 \qquad (S3)$$

1.9.2. Hemolysis assay of 5Nd/Yb/Er DCNPs

A 3 mL sample of mouse blood was centrifuged at 3000 RPM with a saline solution to isolate red blood cells. The erythrocytes were then suspended in PBS at a 1:2 volume ratio. Subsequently, 5Nd/Yb/Er DCNPs solutions at varying concentrations (0 to 100 µg/mL) were mixed with 0.5 mL of the diluted erythrocyte suspension. All mixtures were incubated at 37 °C for 3 to 4 hours. After the incubation, samples were centrifuged at 800 x g for 10 minutes. The absorbance of the supernatant was measured at 540 nm using a BioTek Epoch microplate reader to quantify hemoglobin release. Hemolysis percentage was calculated using the following formula:

$$Hemolysis\ (\%) = \frac{A_{positive} - A_{negative}}{A_{sample} - A_{negative}} \times 100 \qquad (S4)$$

Here, TX100 was used as a positive control, and PBS served as a negative control.

1.9.3. In vitro evaluation of cellular uptake

The 4T1 breast cancer cells were seeded in the glass-bottom dishes at a density of 1 x 10^5 cells per dish, cultured for 24 hours at 37 °C in a humidified 5% CO_2 atmosphere. The medium was then replaced with fresh medium containing different concentrations (0, 10, 50, and 100 µg/mL) of rhodamine B-labelled 5Nd/Yb/Er DCNPs, and the cells were

incubated for different time intervals (1 hour, 2 hours, 4 hours, 8 hours, and 12 hours). AT each time point, cells were washed with PBS, fixed with 4% paraformaldehyde for 15 min, and stained with DAPI for nuclear visualization [16]. Confocal images were acquired using a Nikon Eclipse Ti2 microscope with an emission range between 500 to 570 nm.

1.9.4. In vitro live/dead assay

The 4T1 breast cancer cells were seeded in 6-well plates and cultured for 24 hours until they reached ~70% to 80% confluence. The cells were then treated with different concentrations (0, 50, and 100 μg/mL) of the 5Nd/Yb/Er DCNPs for 8 hours at 37 °C, followed by staining with Calcein-AM and propidium iodide (PI) for 30 min to distinguish live and dead cells. After PBS washing, fluorescence images were captured using a fluorescence microscope [17].

1.9.5. Development of in vivo subcutaneous 4T1 breast tumor-bearing mouse model

Female BALB/c nude mice (5 to 6 weeks old, 17 to 20 g of body weight) were purchased from Guangdong Yuyuan Biotechnology Co., Ltd., China. All the experimental procedures were approved by the Institutional Animal Care and Use Committee of Shenzhen Baoan Women's and Children's Hospital (Approval No. LLSC-2021-03-9-08-KS). The mice were housed under standard conditions at 25 °C and 55% relative humidity, with free access to food and water. For the tumor model establishment, each mouse received a subcutaneous injection of 1 x 10^6 4T1 breast cancer cells dispersed in 100 μL of PBS. In vivo studies were initiated 10 days later, once the tumor volumes reached 50 to 70 mm^3 [18].

1.9.6. NIR-II fluorescence imaging-guided surgery

Female BALB/c nude mice bearing 50 to 70 mm^3 4T1 breast cancer were administered with 5Nd/Yb/Er DCNPs (10 mg/kg of body weight). The mice were placed under a Teledyne NIR-II fluorescence imaging system (Princeton Instruments, USA. Ex, 808 nm; long-pass filter, 1000 nm; exposure time, 1000 ms; power density, 40 mW/cm^2) for surgical navigation in imaging-guided surgery [18]. The tumor was removed 3 hours post-injection until no residual fluorescence signals remained. After that, the mice were monitored for 6-7 days to observe any further lesion growth or any other abnormality, and the mice were humanely euthanized [19].

1.9.7. H&E staining

Tissue samples were collected from the mice after NIR-II fluorescence imaging-guided surgery and were prepared for H&E staining following the standard protocol of BBC Biochemical Company. Briefly, 8 μm frozen sections were fixed in 10% formalin for 30 min, rinsed, and dehydrated through graded ethanol solutions. The sections were stained with hematoxylin and eosin, cleared with xylene, and mounted using Canada balsam. Histological images were acquired using a Nikon Eclipse 90i microscope [2].

1.9.8. Blood biochemistry studies

The blood samples were obtained from the retro-orbital sinus of anesthetized BALB/c mice bearing 4T1 breast cancer in each treatment group (PBS and DCNPs) and collected into heparinized tubes. The samples were centrifuged at 3 x 10^3 g for 10 min at 4 °C to separate the plasma, which was then aliquoted and stored at -80 °C prior to analysis. Biochemical parameters were analyzed following standard protocols on a clinical chemistry analyzer using commercial assay kits. Creatinine and uric acid levels were determined by enzymatic colorimetric methods, while the activities of alanine aminotransferase (ALT), aspartate aminotransferase (AST), and alkaline phosphatase (ALP) were measured using kinetic UV-based assays in accordance with IFCC-recommended procedures. The results are expressed in standard units, with creatinine and uric acid reported in μmol/L and enzyme activities in U/L [20].

1.9.9. Statistical analysis

All experiments were evaluated as mean values ± SD of at least three tests. A one-way analysis of variance (ANOVA) program was used to evaluate the statistical significance. The alpha value is 0.05, the p value $^{*}p < 0.05$, $^{**}p < 0.01$, $^{***}p < 0.001$, $^{****}p < 0.0001$, and n.s. indicates no significance.

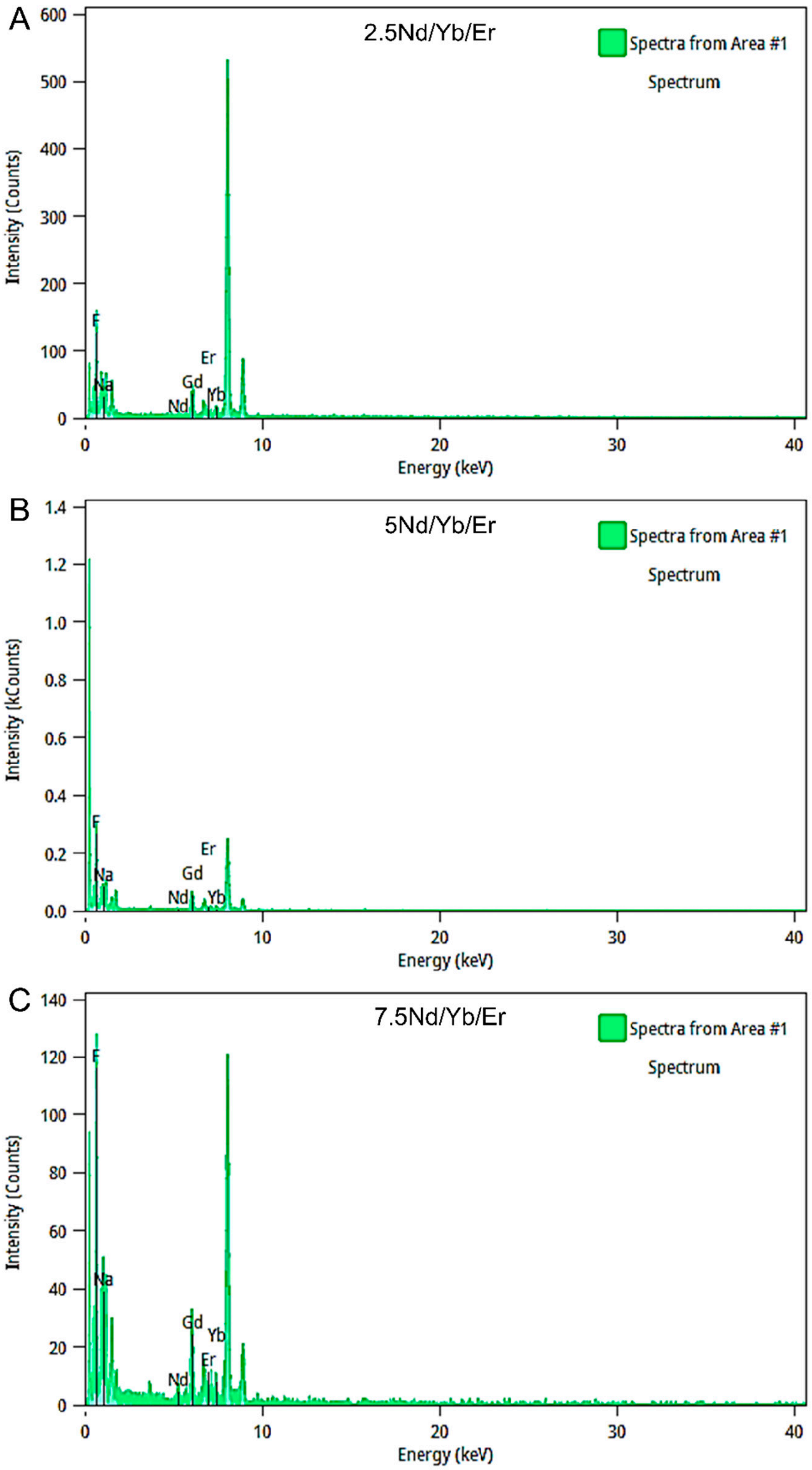

Figure S1. The EDX spectra of **(A)** 2.5Nd/Yb/Er, **(B)** 5Nd/Yb/Er, and **(C)** 7.5Nd/Yb/Er demonstrating the presence of all the component elements (Na, F, Gd, Yb, Er, Nd).

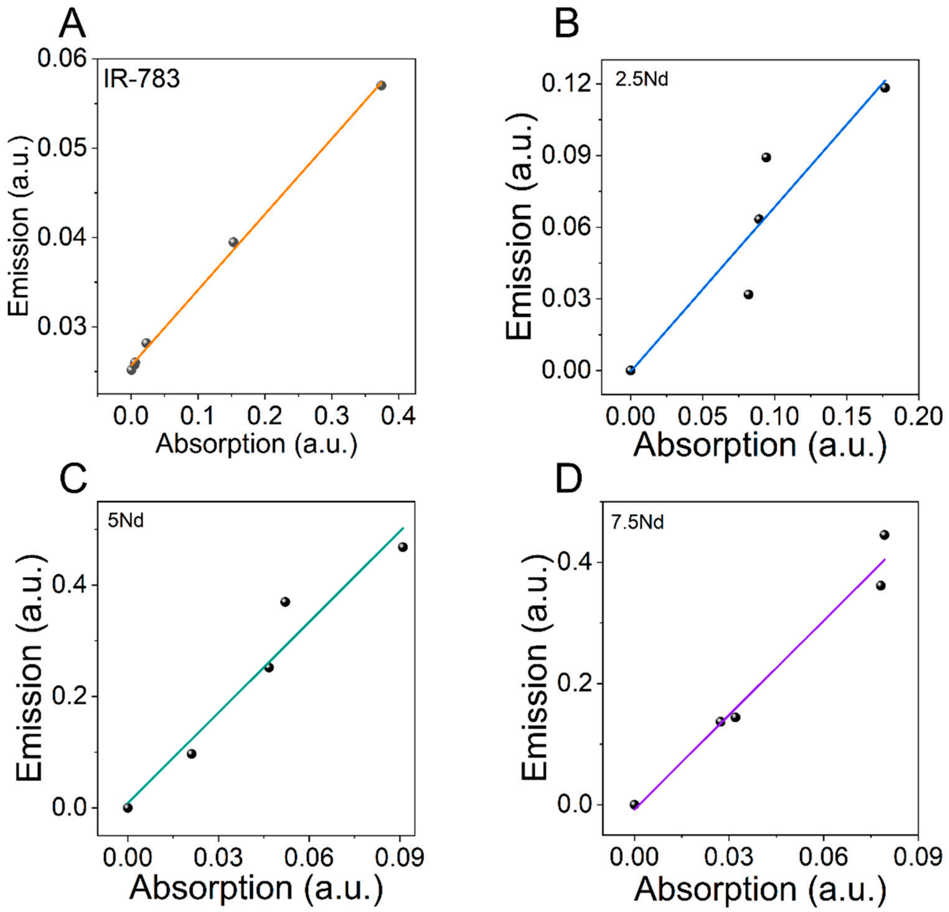

Figure S2. Emission *vs.* absorbance graphs for **(A)** IR-783 dye, **(B)** 2.5Nd/Yb/Er, **(C)** 5Nd/Yb/Er, and **(D)** 7.5Nd/Yb/Er DCNPs.

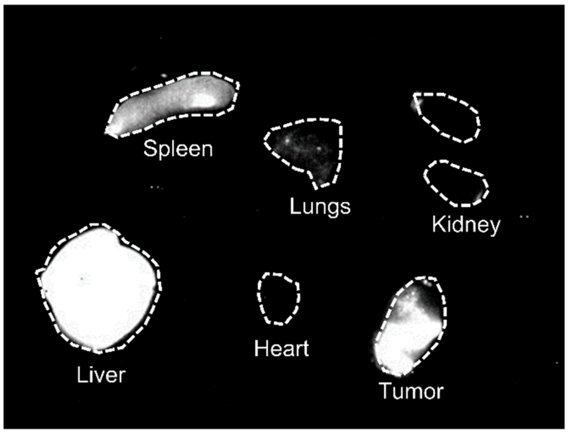

Figure S3. *Ex vivo* NIR-II FL image of the tumor and major body organs demonstrates the biodistribution of the sample in tumor bearing mice model.

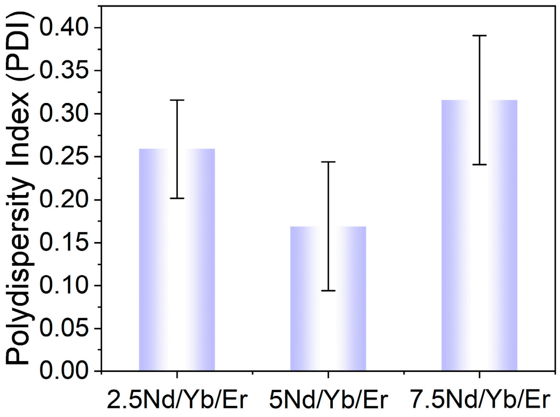

Figure S4. Polydispersity index demonstrates the aqueous colloidal stability of different variants of DCNPs.

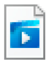

Video S1.mp4

Video S1. Demonstration of survival of the subcutaneous breast tumor-bearing mice after the NIR-II FL imaging-guided surgery.

Reference

1. Roy, S.; Fan, Z.; Ullah, Z.; Madni, M.; Shyamal, S.; Roy, J.; Bag, N.; Sun, L.; Qian, X.; Zhang, Y. Ultrasound-powered piezoelectric hydrogel enables dual Piezodynamic-Chemodynamic therapy and immunomodulation against bacteria-infected burn wounds. *Nano Energy* **2025**, *146*, 111535.
2. Ullah, Z.; Roy, S.; Hasan, I.; Madni, M.; Gong, T.; Roy, J.; Sau, A.; Yan, Y.; Soe, S.K.; Zhang, Y. NIR-II Fluorescence and Magnetic Resonance Imaging-Guided Efficient Piezodynamic Therapy of Subcutaneous Glioblastoma with a Biomimetic Nanoplatform Containing Defect-Engineered Piezoelectric Shell and Downconversion Nanocore. *Small* **2025**, *21*, e10697.
3. Roy, S.; Wang, S.; Ullah, Z.; Hao, H.; Xu, R.; Roy, J.; Gong, T.; Hasan, I.; Jiang, W.; Li, M. Defect-Engineered Biomimetic Piezoelectric Nanocomposites With Enhanced ROS Production, Macrophage Re-polarization, and Ca2+ Channel Activation for Therapy of MRSA-Infected Wounds and Osteomyelitis. *Small* **2025**, *21*, 2411906.
4. Bag, N.; Roy, J.; Sau, A.; Mondal, I.; Chatterjee, A.; Ullah, Z.; Madni, M.; Mondal, D.; Bhandary, S.; Roy, S. Lanthanide-induced defect-engineered BTO nanoparticles as ultrasound-driven reusable piezocatalysts for degradation of organic dyes, antibiotics, and bacterial pathogens for comprehensive wastewater treatment. *Separation and Purification Technology* **2025**, *382*, 135839.
5. Mondal, D.; Roy, S.; Sau, A.; Roy, J.; Bag, N.; Ullah, Z.; Ghosh, S.; Gong, T.; Madni, M.; Chakraborty, I. Chitosan cloaked MWCNT-kaolinite bio-nanocomposite for energy generation and ultrasound driven ROS induced degradation of organic dyes and pathogen. *Ceramics International* **2025**, *51*, 45744-45754.
6. Yang, Y.; Hu, X.; Yang, Z.; Huang, W. Insights into molecular lanthanide complexes: construction, properties and bioimaging and biosensing applications. *Advanced Functional Materials* **2025**, *35*, 2412970.
7. Kong, X.; Liang, J.; Lu, M.; Zhang, K.; Zhao, E.; Kang, X.; Wang, G.; Yu, Q.; Gan, Z.; Gu, X. A NIR-II Organic Dendrimer with Superb Photothermal Performance

Based on Electron-Donor Iteration for Photothermal Immunotherapy. *Advanced Materials* **2024**, *36*, 2409041.

8. Chen, Y.; Chen, S.; Yu, H.; Wang, Y.; Cui, M.; Wang, P.; Sun, P.; Ji, M. D–A type NIR-II organic molecules: strategies for the enhancement fluorescence brightness and applications in NIR-II fluorescence imaging-navigated photothermal therapy. *Advanced Healthcare Materials* **2022**, *11*, 2201158.
9. Yang, Y.; Jiang, Q.; Zhang, F. Nanocrystals for deep-tissue in vivo luminescence imaging in the near-infrared region. *Chemical Reviews* **2023**, *124*, 554-628.
10. Li, C.; Pang, Y.; Xu, Y.; Lu, M.; Tu, L.; Li, Q.; Sharma, A.; Guo, Z.; Li, X.; Sun, Y. Near-infrared metal agents assisting precision medicine: from strategic design to bioimaging and therapeutic applications. *Chemical Society Reviews* **2023**, *52*, 4392-4442.
11. Tang, Y.; Li, Y.; He, C.; Wang, Z.; Huang, W.; Fan, Q.; Liu, B. NIR-II-excited off-on-off fluorescent nanoprobes for sensitive molecular imaging in vivo. *Nature Communications* **2025**, *16*, 278.
12. Yang, A.; Wang, Y.; Feng, Q.; Fatima, K.; Zhang, Q.; Zhou, X.; He, C. Integrating Fluorescence and Magnetic Resonance Imaging in Biocompatible Scaffold for Real-Time Bone Repair Monitoring and Assessment. *Advanced Healthcare Materials* **2024**, *13*, 2302687.
13. Zhang, Q.; Zhou, D.; Fang, G.; Lu, H.; Zeng, J.; Gu, Z. Cell-Derived Biomimetic 2D Nanoparticles to Improve Cell-Specific Targeting and Tissue Penetration for Enhanced Magnetic Resonance Imaging. *Advanced Materials Interfaces* **2022**, *9*, 2101914.
14. Wu, J.; Zhou, X.; Tsang, C.Y.; Mei, Q.; Zhang, Y. Bioengineered nanomaterials for dynamic diagnostics in vivo. *Chemical Society Reviews* **2025**, *54*, 5470-5515.
15. Ma, X.; Zhang, M.J.; Wang, J.; Zhang, T.; Xue, P.; Kang, Y.; Sun, Z.J.; Xu, Z. Emerging biomaterials imaging antitumor immune response. *Advanced Materials* **2022**, *34*, 2204034.
16. Singh, B.; Maharjan, S.; Pan, D.C.; Zhao, Z.; Gao, Y.; Zhang, Y.S.; Mitragotri, S. Imiquimod-gemcitabine nanoparticles harness immune cells to suppress breast cancer. *Biomaterials* **2022**, *280*, 121302.

17. Hu, C.; He, S.; Lee, Y.J.; He, Y.; Kong, E.M.; Li, H.; Anastasio, M.A.; Popescu, G. Live-dead assay on unlabeled cells using phase imaging with computational specificity. *Nature communications* **2022**, *13*, 713.
18. Fang, C.; Liu, Y.; Zhang, B.; Liu, T.; Hu, X.; Li, B.; Gan, Y.; Huang, C.; Xue, P.; Zhang, S. A Photothermal Nano-Switch for Tumor-Selective Wnt Hyperactivation in Vivo. *Angewandte Chemie* **2025**, *137*, e202506666.
19. Ye, J.; Hu, P.; Zhang, R.; Zhou, L.; Luo, Z.; Chen, Y.; Ruan, S.; Zhu, M.; Ding, H.; Qian, Y. Targeting Hyperglycemic Bone Pre-Metastatic Niche for Breast Cancer Bone Metastasis Therapy. *Advanced Science* **2025**, *12*, e04924.
20. Yang, H.; Liu, Y.; Li, M.; Zhang, X.; Zhao, Y.; Luo, Z. Photo-Controlled MUC1 Ablation via Steric Locking of Turn-On Reactors Enhances Pyroptosis-Immunotherapy of Triple Negative Breast Cancer. *Advanced Materials* **2025**, *38*, e04460.
